# Tumor budding and laminin5-γ2 in squamous cell carcinoma of the external auditory canal are associated with shorter survival

**DOI:** 10.1186/s40064-015-1620-4

**Published:** 2015-12-24

**Authors:** Yasuko Okado, Mikiko Aoki, Makoto Hamasaki, Kaori Koga, Takayuki Sueta, Hideki Shiratsuchi, Yoshinao Oda, Takashi Nakagawa, Kazuki Nabeshima

**Affiliations:** Department of Pathology, Fukuoka University School of Medicine and Hospital, 7-45-1 Nanakuma, Jonan-ku, Fukuoka, 814-0180 Japan; Department of Otorhinolaryngology, Fukuoka University School of Medicine and Hospital, Fukuoka, Japan; Department of Otorhinolaryngology, Hamanomachi Hospital, Fukuoka, Japan; Department of Otorhinolaryngology, Graduate School of Medical Sciences, Kyushu University, Fukuoka, Japan; Department of Anatomic Pathology, Graduate School of Medical Sciences, Kyushu University, Fukuoka, Japan

**Keywords:** Squamous cell carcinoma, External auditory canal, Tumor budding, Laminin 5-γ2

## Abstract

Squamous cell carcinoma (SCC) of the external auditory canal (EAC) is rare, usually presents at an advanced stage, and is a more aggressive tumor with poor prognosis. The University of Pittsburgh TNM staging system commonly used in prognostication is not perfect, and more accurate biomarkers predicting prognosis are needed. Tumor budding is an established negative prognostic factor at the invasive front in colorectal cancer. Moreover, immunohistochemical studies showed that laminin 5-γ2 (Ln5-γ2) is expressed at the invasive front in tumor or tumor budding cells. We assessed the prognostic significance of tumor budding and Ln5-γ2 expression by performing Ln5-γ2 immunohistochemistry and evaluated the degree of tumor budding in pre-treatment biopsy specimens, and investigated their correlations to clinicopathological parameters in patients with SCC of the EAC. Patients whose tumors had high budding grade and Ln5-γ2 expression had significantly shorter survival times. Budding grade was significantly correlated with Ln5-γ2 expression. Multivariate analysis revealed that high budding grade predicted poorer prognosis regardless of disease stage. Our results suggested that budding grade and Ln5-γ2 expression can be used as indicators of poor prognosis in patients with SCC of the EAC.

## Background

Squamous cell carcinoma (SCC) of the external auditory canal (EAC) is an aggressive malignancy with a poor prognosis. The reported incidence is less than six cases per million per year, accounting for 0.2 % of all tumors of the head and neck, with a reported 5-year disease-specific survival ranging from 19 to 48 % (Moffat et al. 2000; Bacciu et al. 2013; Prasad et al. 2014; Masterson et al. 2014). Because of the rarity of this tumor, it has been difficult for any single institution to analyze the clinical data and formulate an optimal evaluation and treatment strategy. Another impediment to the study of SCC of the EAC is the lack of a universally accepted staging system (Bacciu et al. 2013; Prasad et al. 2014; Masterson et al. 2014; Nakagawa et al. 2006). Currently, there is no recognized stand-alone American Joint Committee on Cancer (Chicago, IL, USA) or International Union Against Cancer (Geneva, Switzerland) staging system for SCC of the EAC (Prasad et al. 2014). A revised University of Pittsburgh (Pittsburg, PA, USA) staging system was proposed in the year 2000 (Moody et al. 2000). This system is the classification tool that is commonly used for prognosis and treatment decisions in patients with SCC of the EAC (Bacciu et al. 2013; Prasad et al. 2014; Masterson et al. 2014; Nakagawa et al. 2006; Moody et al. 2000). However, this tool does not correctly classify every patient (Prasad et al. 2014; Mazzoni et al. 2014); although the number is small, there are some patients with tumors that recur locally despite their categorization as low-risk and, conversely, patients with longer survival times despite their categorization as high-risk, based on the TNM system (Prasad et al. 2014). The failure of TNM staging to serve as a reliable prognostic system for patients with intermediate-stage tumors may be overcome by considering morphologic, molecular, or treatment-related factors to stratify patients more precisely into different risk categories. Thus, search for biomarkers that reflect the biologic characteristics of SCC of the EAC and treatment outcome prediction is crucial (Marioni et al. 2012, 2013).

Tumor budding/sprouting is a histological feature at the invasive front of a tumor and an established negative prognostic factor when present in resected colorectal cancer and preoperative rectal cancer biopsy specimens (Morodomi et al. 1989; Hase et al. 1993; Ueno et al. 2002; Zlobec and Lugli 2010; Mitrovic et al. 2012; Rogers et al. 2014; Satoh et al. 2014). The concept of tumor budding/sprouting was first described in the 1950s by Imai, who postulated that the presence of “sprouting” at the invasive edge of a carcinoma indicates a more rapid tumor growth rate (Imai 1960). Tumor budding is thought to represent an active invasive form of cancer; specifically, it is the isolation and mobilization of cancer cells from a main tumor mass during the early stages of tumor invasion (Hase et al. 1993). Tumor cells that have undergone the epithelial-to-mesenchymal transition (EMT) are indicated histologically by the presence of tumor budding (Mitrovic et al. 2012). Tumor budding is predictive of lymph node metastasis, vascular and lymphatic invasion, non-responsiveness to neoadjuvant chemo-radiotherapy, distant metastasis, local recurrence, and a poor prognosis (Morodomi et al. 1989; Ueno et al. 2002; Koike et al. 2008; Brown et al. 2010; Sarioglu et al. 2010; Zlobec and Lugli 2010; Luo et al. 2012; Mitrovic et al. 2012; Rogers et al. 2014; Satoh et al. 2014). A high level of tumor budding/sprouting is associated with a poorer prognosis in several human solid cancers, including some types of SCC, e.g., esophageal SCC (Koike et al. 2008; Brown et al. 2010), laryngeal carcinoma (Sarioglu et al. 2010), and nasopharyngeal carcinoma (Luo et al. 2012). For head and neck SCC, the importance of invasion pattern for prognosis has been extensively addressed since seventies (Willen et al. 1975; Anneroth et al. 1987; Bryne et al. 1992, 1998). The studies have shown that it is not the differentiation grade but the invasion pattern that is important for assessment of clinicopathological aggressiveness. Particularly, the invasion pattern classified as Grade or Score 4, which referred to neoplasms with a marked diffuse, widespread cellular invasion of the neoplasm in single neoplastic cells or in small groups of cells, corresponds to tumor budding mentioned herein (Anneroth et al. 1987). More recently, a non-cohesive invasive front is reported to be an indicator of metastasis in the SCC of the ear auricle, which is known to metastasize to the regional lymph nodes more frequently than SCC at other sites (Clark et al. 2010).

Previously, our laboratory showed that laminin 5-γ2 (Ln5-γ2) has a role in invasion of cutaneous SCC (Hamasaki et al. 2011). We also reported that even in the early stages of SCC of the EAC, diffuse expression of Ln5-γ2 is associated with a poor prognosis (Nakagawa et al. 2013). Laminin 5, a heterotrimer composed of three different Laminin chains (α3-, β3-, γ2-), is the major component of the basement membrane in most adult tissues (Nguyen et al. 2000). One of the chains, Ln5-γ2, is a specific marker for invasive tumors because it is frequently expressed as a monomer in several types of malignant tumors (Nguyen et al. 2000; Koshikawa et al. 2008). Several immunohistochemical studies have found that Ln5-γ2 expression is observed exclusively in the cytoplasm of budding cells at the invasive front in many types of human cancers, such as colon, and breast cancer, and SCC of the esophagus, cervix, and skin (Pyke et al. 1995; Yamamoto et al. 2001; Hamasaki et al. 2011).

To our knowledge, the degree of tumor budding in SCC of the EAC has not yet been described. The aim of this study was to assess the degree of tumor budding and its correlation to Ln5-γ2. We also performed an immunohistochemical analysis of Ln5-γ2 in pre-treatment ear cancer biopsy specimens to investigate the prognostic significance of Ln5-γ2 expression.

## Methods

### Patients

We retrospectively reviewed clinicopathological data for 46 patients with primary SCC of the EAC, for whom the pre-treatment tissue specimens were available, and who were treated by the same strategy at the Department of Otorhinolaryngology, Kyushu University Hospital (Fukuoka, Japan) from January 1998 to March 2006 and at the Department of Otorhinolaryngology, Fukuoka University Hospital (Fukuoka, Japan) from April 2006 to December 2014. Patients who underwent chemotherapy or radiation prior to biopsy, patients for whom specimens were not available, or patients who failed to follow our treatment plan, were excluded from the study. As long as the provisions for maintaining patient anonymity are followed, tissues from biopsy samples can be used for research purposes per a standard treatment agreement with Kyushu University and Fukuoka University Hospitals. The study protocol was approved by the Institutional Review Board (The Ethics Committee) of Kyushu University (No. 26-185) and Fukuoka University (No. 12-7-13) Hospitals. Clinical stage was determined using the staging protocol proposed by the University of Pittsburgh (Moody et al. 2000). Before treatment, the extent of disease was estimated in all patients by physical examination and imaging studies [Computed tomography (CT), and magnetic resonance imaging (MRI)]. The invasion of the canal wall of the internal carotid artery, jugular bulb, otic capsule, and dura mater was determined by CT, and the extension to the soft tissue, petrous apex, middle ear, mastoid, temporomandibular joint, and parotid gland was distinguished by MRI. CT and MRI scans were routinely conducted every 6 months, for 3 years, after therapy. After 3 years, CT and MRI were performed annually. The surgical or post-irradiation scarring was often indistinguishable from residual or recurrent disease. An absence of any signs of enlargement of a space-occupying lesion, was designated as “no evidence of disease”. The follow-up period for the complete series ranged from 4 to 66 months (median, 34 months).

### Tissue samples and immunohistochemistry (IHC)

Biopsy specimens were fixed in 10 % formalin and processed into paraffin blocks. Paraffinized tissue blocks were sectioned (4-μm thickness), deparaffinized, and hydrated in descending alcohol dilutions. For anti-cytokeratin (CK) antibody staining, sections were immersed in 3 % hydrogen peroxide in water for 10 min at room temperature (RT) to block endogenous peroxidase activity, and heated in 10 mM ethylenediaminetetraacetic acid (EDTA) buffer (pH 8.0) in a microwave (700 W) for 10 min to retrieve epitopes before staining. For anti-Ln5-γ2 antibody staining, sections were immersed in 0.05 % protease XXIV (Sigma-Aldrich, Tokyo, Japan) for 15 min at RT to block endogenous peroxidase activity. The sections were incubated with anti-human CK monoclonal antibody (AE1/AE3, Dako; 1:200), or anti-human Ln5-γ2 monoclonal antibody (D4B5, Temecula, CA, USA; 1:200), for 1 h at RT (for CK), or for 1 h at 37 °C (for Ln5-γ2). The sections were then washed in Tris-buffered saline (TBS) and incubated for 30 min at RT with EnVision reagent conjugated horseradish peroxidase (Dako; for CK), or Histofine reagent conjugated to alkaline phosphatase (Nichirei Bioscience; for Ln5-γ2). Immunoreactive proteins were visualized with 3,3′-diamino-benzidine (DAB; Dako; for CK), or New Fuchsin substrate kit (Nichirei Bioscience, Tokyo, Japan; for Ln5-γ2), followed by counterstaining with Mayer’s hematoxylin (for CK), or methylgreen (for Ln5-γ2). The stained sections were evaluated semi-quantitatively by two independent observers who were blinded to the clinical data.

### Grading for tumor budding/sprouting

**Fig. 1:**
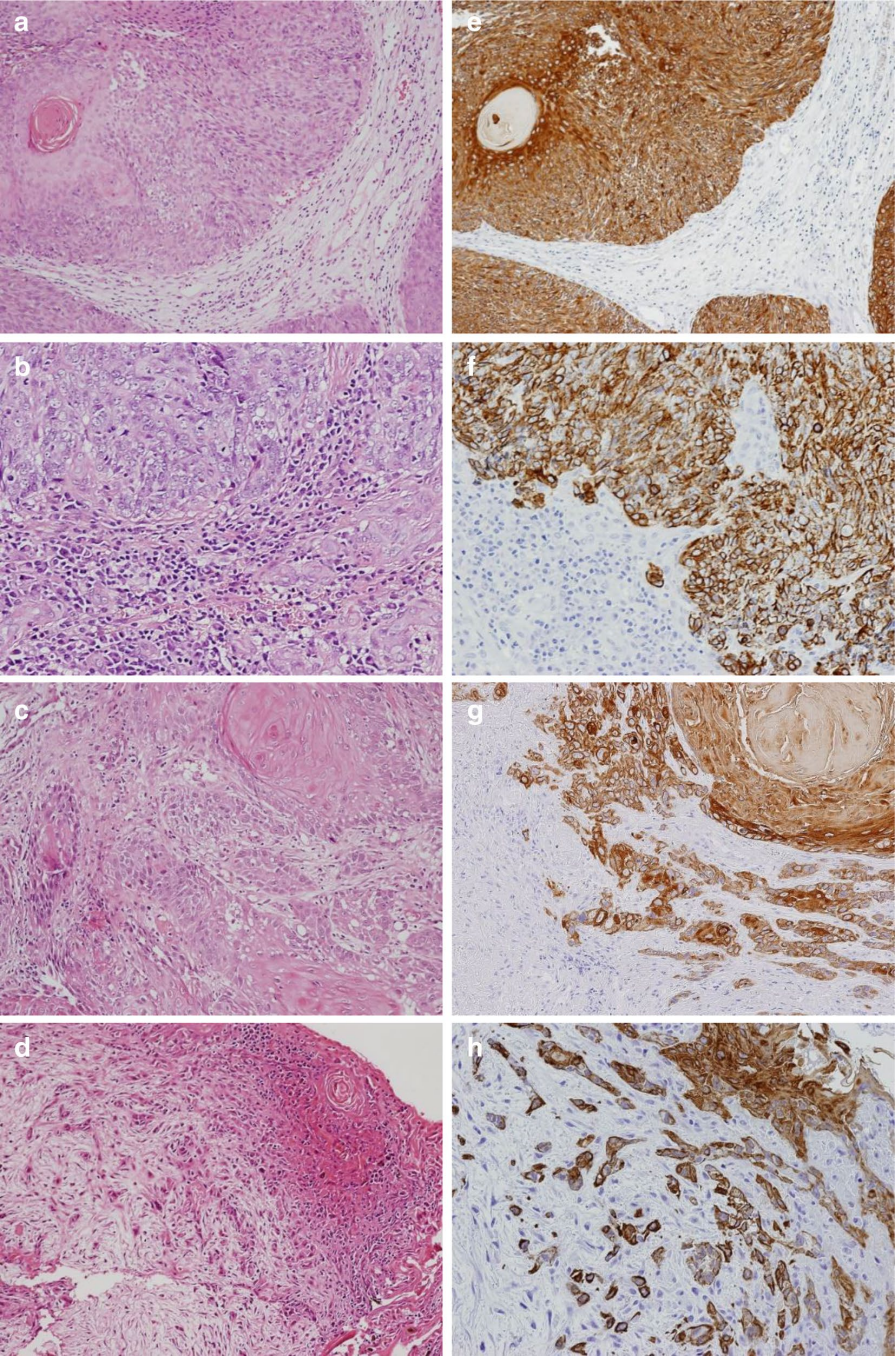
Hematoxylin-eosin staining of SCC of the EAC showing tumor budding/sprouting (**a**–**d**) and cytokeratin immunohistochemistry (CK-IHC) for tumor budding/sprouting (**e**–**h**) at the invasive front. *Inset* in **a**, **e** shows absence of tumor budding; budding grade 0, the others shows presence of tumor budding; **b**, **f** shows budding grade 1, **c**, **g** shows budding grade 2, and **d**, **h** shows budding grade 3

Based on the Japanese Classification of Colorectal Carcinoma (JCCC) (Rectum JSfCotCa 2013), we defined tumor budding/sprouting as a cancer cell nest consisting of five or fewer cells that infiltrated the interstitium at the invasive margin of the cancer. After selecting an area in which budding/sprouting was most intensive, the buds were counted in a field measuring 0.785 mm^2^ through a 20× objective lens (WHK 10× ocular lens; Olympus, Tokyo, Japan). Depending on the number of buds, we used the modified grading system proposed by Satoh et al. (2014) for estimation of tumor budding: Grade 0, no budding (Fig. 1a, e); Grade 1, 1–4 buds (Fig. 1b, f); Grade 2, 5–9 buds (Fig. 1c, g); Grade 3, ≥10 buds (Fig. 1d, h). We used this grading system to evaluate tumor budding in H&E- (Fig. 1a–d) and CK-immunostained (Fig. 1e–h) sections. The degree of tumor budding was classified as low grade or high grade, corresponding to 0–9 (grades 0, 1, and 2) or ≥10 budding foci (grade 3) in one field, respectively, according to Ueno et al. (2002). This grading system has been proven to be have prognostic significance, is easily applied, and is frequently used in tumor budding studies (Mitrovic et al. 2012).

### Immunohistochemical analysis of Ln5-γ2

**Fig. 2:**
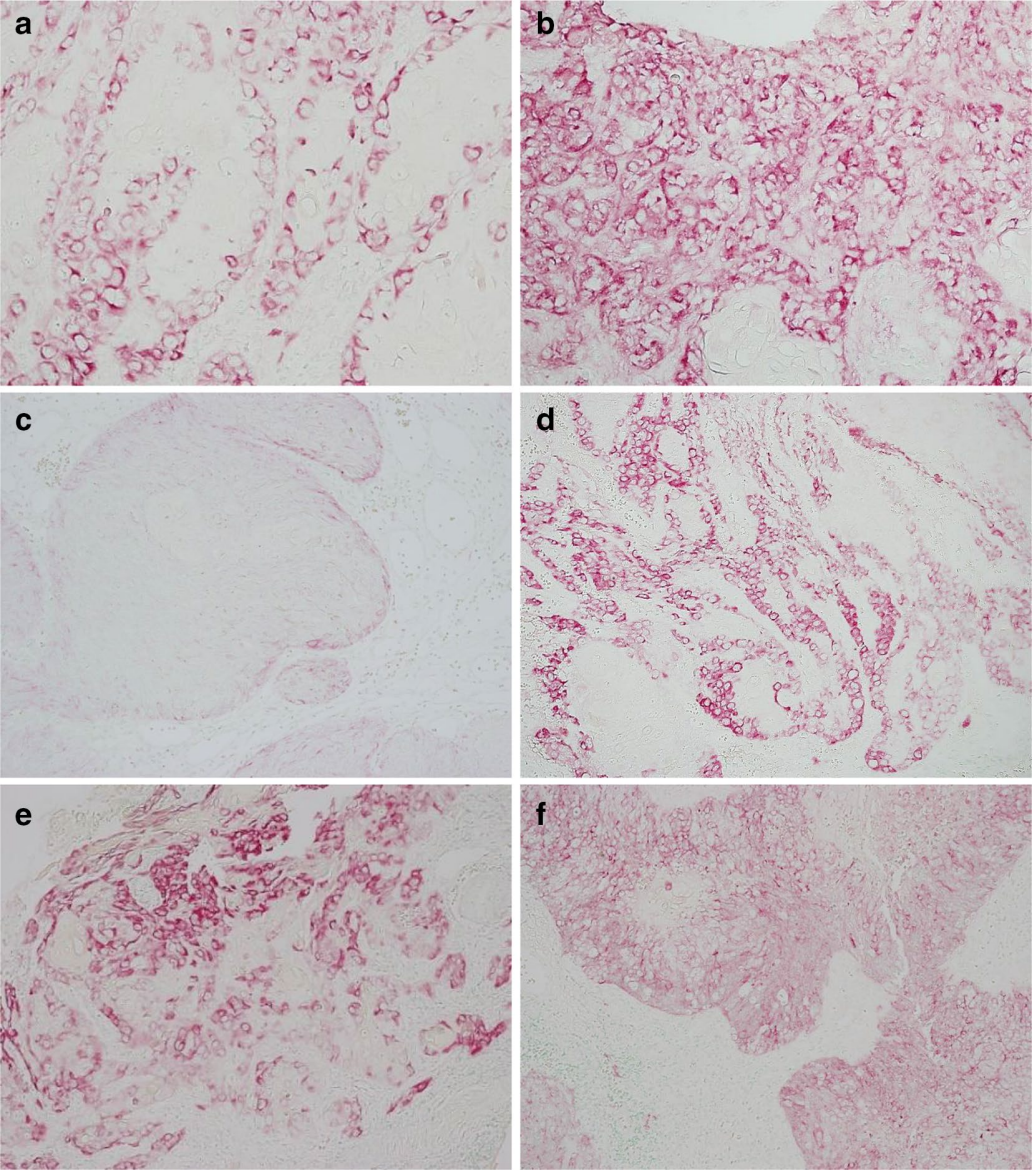
Immunohistochemical analysis of Ln5-γ2 expression in SCC of the EAC. **a** Marginal expression pattern. **b** Diffuse expression pattern. The expression level is scored as 1+ to 4+ based on the percentage of positively stained tumor cells. **c** 1+: 0–24 %, **d** 2+: 25–49 %, **e** 3+: 50–74 %, **f** 4+: 75–100 %

Immunoreactivity for Ln5-γ2 was observed as cytoplasmic staining in carcinoma cells. The immunostaining result was considered to be negative if <10 % of the tumor cells were stained. In specimens considered to have a positive result, the tumor staining pattern was assessed as marginal (Fig. 2a) or diffuse (Fig. 2b), and was quantitated on a scale from 1 to 4 based on the percentage of positively-stained tumor cells. The scale was as follows: 1+, 10–24 % positive (Fig. 2c); 2+, 25–49 % positive (Fig. 2d); 3+, 50–74 % positive (Fig. 2e); and 4+, 75–100 % positive (Fig. 2f). Each tumor was then classified as low-expression (1+ and 2+) or high-expression (3+ and 4+).

### Statistical analysis

The relationships between several clinicopathological parameters and the results of budding assessments and IHC were evaluated using the Student’s-*t* test for continuous variables, and Fisher’s exact test for categorical variables. Disease-specific survival curves were plotted by the Kaplan–Meier method, and *P* values were calculated using the log-rank test. A univariate analysis was performed for each clinicopathological parameter. Multivariate analysis and the Cox proportional-hazard model were used to determine the independent prognostic factors. A *P* value <0.05 was considered statistically significant. All data analyses were conducted with JMP10.0.2 (SAS Institute Inc., Cary, NC, USA) for Windows.

## Results

### Clinical and histopathological findings

Table 1 summarizes the clinicopathological characteristics of 46 patients [25 male and 21 female; age range, 38–86 years (mean = 62.5)]. The median follow-up period was 34.6 months (range 4–66 months). The TNM stages for a majority of tumors was stage IV [25 (54.3 %)]. The proportion of distant metastasis was low (4.3 %), similar to that reported in previous studies (Moffat et al. 2000; Moody et al. 2000; Nakagawa et al. 2006; Bacciu et al. 2013; Prasad et al. 2014; Masterson et al. 2014). The proportion of patients in which the cancer had spread to the lymph nodes was 21.7 %, slightly higher than that in previous studies (ranging from 10 to 15 %) (Moffat et al. 2000). Because all the metastatic nodes were in an advanced stage according to the staging protocol proposed by University of Pittsburgh, we considered N1, N2a, and N2b as N+. The presence of lymphovascular invasion could not be assessed because of the size of the biopsy specimens. Lateral temporal bone resection was achieved in 15 cases, subtotal temporal bone resection was achieved in 16 cases, and 15 cases received only chemo-radiotherapy. Of the 31 (69.6 %) patients who underwent surgery, postoperative data was available for 21 (65.6 %) patients and 6 (29 %) patients had postoperative local recurrence. Of these 21 patients, 18 (85.7 %) had received neoadjuvant chemo-radiotherapy. Of these patients, we assessed the effect of therapy using surgical tissue specimens, according to the General Rules for Clinical Studies on Head and Neck Cancer (Cancer JSfHaN 2012). The effect of therapy was classified as one case (5.6 %) with a grade 0, one (5.6 %) with a grade 1, 9 (50.0 %) with a grade 2, and 7 (38.9 %) with a grade 3, response.

**Table 1:** Clinicopathological features of 46 patients with SCC of EAC and their correlation to tumor growth pattern and budding grade

	All	Tumor budding			Budding grade		
	n (%)	Absent 13 (28.3)	Present 33 (71.7)	*P* value	Low 39 (84.8)	High 7 (15.2)	*P* value
Mean age [years, (sd)]	62.5 (±11.4)	65.0 (±3.1)	61.5 (±1.9)	0.358	63.1 (±1.8)	59.3 (±0.4.3)	0.426
M:F	21:25 (46:54)	8:5 (62:38)	17:16 (52:48)	0.743	21:18 (54:46)	4:3 (57:43)	1.000
T							
T1	1 (2.2)						
T2	12 (26.1)	4 (30.8)	9 (27.3)		11 (28.2)	2 (28.6)	
T3	9 (19.5)						
T4	24 (52.2)	9 (69.2)	24 (72.7)	1.000	28 (84.8)	5 (71.4)	1.000
N							
N0	36 (78.3)	11 (84.6)	25 (75.8)		31 (79.5)	5 (71.4)	
N+	10 (21.7)	2 (15.4)	8 (24.2)	0.700	8 (20.5)	2 (28.6)	0.636
M							
M0	44 (95.7)	13 (100)	31 (94.0)		38 (97.4)	6 (85.7)	
M+	2 (4.3)	0 (0)	2 (6.0)	1.000	1 (2.6)	1 (14.3)	0.284
TNM stage							
Stage I	1 (2.2)						
Stage II	10 (21.7)	4 (30.8)	7 (21.2)		10 (25.6)	1 (14.3)	
Stage III	10 (21.7)						
Stage IV	25 (54.4)	9 (69.2)	26 (78.8)	0.702	29 (74.4)	6 (85.7)	1.000
Differentiation							
Well	28 (60.9)	7 (53.8)	21 (63.6)		26 (66.7)	2 (28.6)	
Moderate	15 (32.6)	6 (46.2)	9 (27.3)		11 (28.2)	4 (57.1)	
Poor	3 (6.5)	0 (0)	3 (9.1)	0.309	2 (5.1)	1 (14.3)	0.157
Recurrence	21						
Free	15 (71.4)	4 (80.0)	11 (68.8)		15 (78.9)	0 (0)	
Recurrence	6 (28.6)	1 (20.0)	5 (31.2)	1.000	4 (21.1)	2 (100)	0.071
Effect of therapy	18						
Grade 0–2	11 (61.1)	1 (25.0)	10 (71.4)		9 (60.0)	2 (66.7)	
Grade 3	7 (38.9)	3 (75.0)	4 (28.6)	0.245	6 (40.0)	1 (33.3)	1.000

*M* male, *F* female, *MCF* middle cranial fossa

### Tumor budding in pre-treatment biopsy samples

Tumor budding was first assessed in H&E-sections (Fig. 1a–d). However, assessment was sometimes difficult because of the presence of reactive fibroblasts or macrophages in the stroma surrounding the invasive front. Therefore, we assessed budding carcinoma cells based on CK-IHC data (Fig. 1e–h), according to a method previously reported by Satoh et al. (2014).

The results for the budding data grading indicated that 13 (28.3 %) patients had tumors graded as 0, 20 (43.5 %) patients had grade 1 tumors, 6 (13.0 %) patients had grade 2 tumors, and 7 (15.2 %) patients had grade 3 tumors. Thirty-nine (84.8 %) patients had low budding grade and 7 (15.2 %) patients had high budding grade tumors. There was no significant association between tumor budding and the clinicopathological parameters (Table 1).

### Expression of Ln5-γ2 and clinicopathological correlations

Immunoreactivity for Ln5-γ2 was observed in all SCC of the EAC cases in this study. All tumors demonstrated cytoplasmic and membranous staining. The expression level was as follows: 20 patients (43.5 %) as 1+, 17 (37.0 %) as 2+, 6 (13.0 %) as 3+, and 3 (6.5 %) as 4+. Thirty-seven (80.4 %) patients had tumors with high expression of Ln5-γ2 (1+, 2+). Nine (19.6 %) patients had low levels (3+, 4 +) of Ln5-γ2 expression. The Ln5-γ2 expression patterns showed significant associations with age, advanced TNM stage (stage III and IV), and poor tumor differentiation (*P* = 0.0161, *P* = 0.0439, and *P* = 0.0200, respectively). High Ln5-γ2 expression levels were significantly associated with older age (*P* = 0.0224).

### Tumor budding and Ln5-γ2 expression

The results for the relationships between tumor budding and Ln5-γ2 expression are summarized in Table 2. There was a significant correlation between presence of tumor budding and high Ln5-γ2 expression levels (*P* = 0.0444). High budding grade correlated significantly with both a diffuse Ln5-γ2 expression pattern and high Ln5-γ2 expression levels (*P* = 0.0458 and *P* = 0.0016, respectively).

**Table 2:** Association of tumor budding and Ln5-γ2 expression

	All n (%)	Tumor budding		*P* value	Budding grade		*P* value
		Absence 13 (28.3)	Presence 33 (71.7)		Low 39 (84.8)	High 7 (15.2)	
Ln5-γ2 expression pattern	46						
Marginal	35 (76.1)	10 (76.9)	25 (75.9)		32 (82.1)	3 (42.9)	
Diffuse	11 (23.9)	3 (23.1)	8 (24.1)	1.000	7 (17.9)	4 (57.1)	0.045
Ln5-γ2 expression level							
Low	37 (80.4)	13 (100)	24 (72.7)		35 (89.7)	2 (28.6)	
High	9 (19.6)	0 (0)	9 (27.2)	0.044	4 (10.3)	5 (71.4)	0.001

*Ln5-γ2* Laminin 5-γ2

### Patients survival

**Fig. 3:**
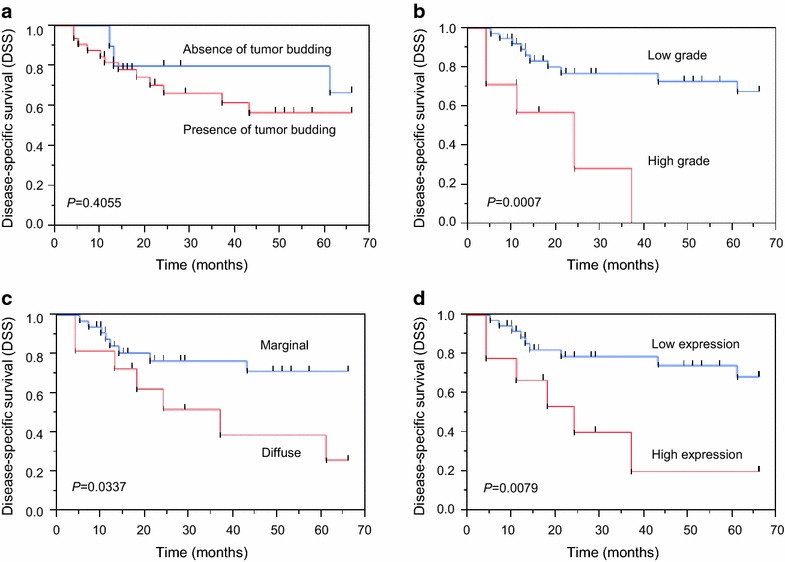
Kaplan–Meier survival curve relating to absent or present of tumor budding (**a**), budding grade (**b**), Ln5-γ2 expression pattern (**c**), and Ln5-γ2 expression level (**d**) in the pre-treatment biopsy specimens from all cases in this study

Disease-specific survival curves, sorted by the presence of tumor budding (Fig. 3a), budding grade (Fig. 3b), and the patterns (Fig. 3c) and levels (Fig. 3d) of Ln5-γ2 expression were analyzed. The patient group with high tumor budding grades had significantly shorter survival times (*P* = 0.0007), but there was no statistically significant difference between the groups in terms of the presence of tumor budding (*P* = 0.4055). The patient groups with a diffuse Ln5-γ2 expression pattern and high Ln5-γ2 expression levels also had significantly shorter survival times (*P* = 0.0337 and *P* = 0.0079, respectively).

**Fig. 4:**
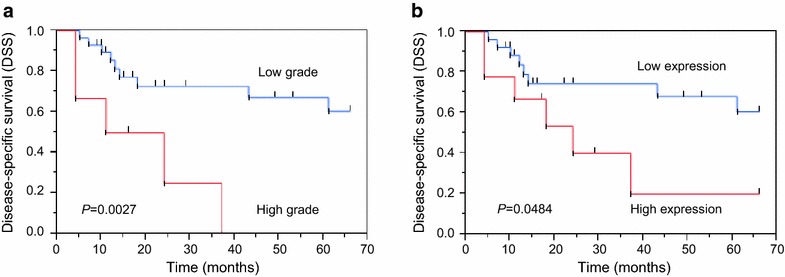
Kaplan–Meier survival curve relating to budding grade (**a**) and Ln5-γ2 expression level (**b**) in the pre-treatment biopsy specimens from only advanced stage (stage III and IV) cancer patients

We also analyzed disease-specific survival in cases with only advanced stages (stage III and IV) of SCC of the EAC, sorted into groups based on the budding grade (Fig. 4a) and Ln5-γ2 expression levels (Fig. 4b). The patient groups with high budding grades and high Ln5-γ2 expression levels had significantly shorter survival times (*P* = 0.0027 and *P* = 0.0484, respectively), even when the analysis was restricted to only those cases with advanced stage disease.

Univariate and multivariate analyses of clinicopathological predictors of disease-specific survival in these cases of SCC of the EAC were also performed. The results indicated that advanced age, patients treated without surgery and high budding grades predicted poorer disease-specific survival (Table 3). The high budding grade was an independent prognostic factor for poorer disease-specific survival in patients with stage III and IV cancer (*P* = 0.010) (Table 3).

**Table 3:** Univariate and multivariate analyses of factors predisposing to disease-specific survival in only cases with stage III and IV cancer

Variable	Univariate analysis			Multivariate analysis		
	Hazard ratio	95 % CI	*P* value	Hazard ratio	95 % CI	*P* value
Age ≥ 70 years	3.163	1.038–9.130	0.043	4.243	0.999–17.759	0.050
Sex (male)	1.247	0.432–3.796	0.682	2.515	0.679–10.827	0.168
Poor differentiation	3.891	0.861–13.096	0.073	1.843	0.284–10.948	0.504
Patients treated without surgery	3.710	1.251–11.643	0.018	2.376	0.628–9.456	0.199
High budding grade	4.930	1.449–15.574	0.012	11.914	1.772–96.778	0.010
High Ln5-γ2 expression level	2.829	0.915–8.332	0.069	0.797	0.114–4.486	0.805

## Discussion

To the best of our knowledge, this study was the first to assess tumor budding/sprouting by grade in SCC of the EAC samples. We found that high budding grade was significantly correlated with poor prognosis in patients with SCC of the EAC. The results of a multivariate analysis indicated that high budding grade was an independent poor prognostic factor even in cases with advanced stage disease, although the number of patients analyzed was small particularly when considering the subgroups for statistical analysis (e.g. 7 patients with high budding grade). We also analyzed Ln5-γ2 expression levels, which have been documented in the literature as being associated with tumor budding (Pyke et al. 1995; Sordat et al. 2000; Nabeshima et al. 2006). The Ln5-γ2 expression level was significantly associated with tumor budding and budding grade. High Ln5-γ2 expression was also significantly associated with shorter disease-specific survival.

Tumor budding/sprouting is a poor prognostic indicator in several types of SCCs (Koike et al. 2008; Zlobec and Lugli 2010; Brown et al. 2010). In the head and neck SCC, the tumor frequently shows higher grade of cellular dissociation at the invasive front than the remaining areas of the tumor, and grading of the invasive tumor front or margins is an independent prognostic factor in multivariate survival analysis (Bryne et al. 1992, 1998). In our study, most of the patients with tumor budding had shorter disease-specific survival times. On the contrary, among the patients with early stage disease (stage I and II), those with no tumor budding survived until the end of the follow-up period (66 months). Several reports have described the relationships between clinicopathological parameters and tumor budding. Morodomi et al. (1989) reported that tumor budding may be a prelude to lymphatic invasion. Koike et al. (2008) reported that tumor budding correlates significantly with lymph node metastasis, lymphatic and vessel invasion, and intramural metastasis in SCC of the esophagus. Similarly in the auricular SCC, Clark et al. (2010) reported that a non-cohesive invasive front is an indicator of lymph node metastasis. However, in our study, regional lymph node status was not significantly associated with tumor budding. The small number and proportion (21.7 %) of cases with lymph node metastasis included in our study might have contributed to these differences.

Expression of Ln5-γ2 in budding cells is associated with focal under-expression of the E-cadherin-β-catenin complex in colorectal carcinoma (Nabeshima et al. 2006). The presence of tumor budding is considered to represent the EMT, a process frequently associated with increased expression of molecules relevant to tumor invasion, such as matrix metalloproteinases and Ln5-γ2 in tumor cells, and stimulation of the Wnt signaling pathway (Zlobec and Lugli 2010). One study found that there is a link between Ln5-γ2-mediated budding activity and decreased cell–cell adhesion (Nabeshima et al. 2006). Similarly, our study revealed that Ln5-γ2 expression was significantly correlated with budding grade. The patient groups with high Ln5-γ2 expression levels had a significantly shorter survival time, regardless of the disease stage. These results indicated that there was an association between higher Ln5-γ2 expression and a poor prognosis.

## Conclusion

In conclusion, tumor budding may be an adverse prognostic factor that can help stratify patients into more meaningful risk groups than TNM staging alone, and more importantly, has a potential to guide clinical decision-making. For example, low budding grade enabled us to sort the patient groups into those with better prognosis even in cases of advanced stage diseases. The treatment of patients with advanced-stage SCC of the EAC includes performing an extended en bloc temporal bone resection, which leaves the patients with a functional and a cosmetic deficit. Our results indicated that tumor budding could allow us to perform less invasive surgery for patients with low budding grade tumors. Additionally, tumor budding can be evaluated with relative ease during a routine pathological examination. Because of the strong correlation between Ln5-γ2 expression and tumor budding, we can use immunohistochemical analysis of Ln5-γ2 as an alternative or concurrent parameter when tumor budding cannot be assessed in the biopsy specimen. (e.g., when the sample is too small or does not including the stroma). Although biological or clinical malignancy of tumors depends on not only invasiveness but also proliferative ability of tumor cells, multivariate analysis in this study showed the mode of invasiveness is more important for survival than tumor size, stage, and differentiation in SCC of the EAC. Further studies are now in practice in our laboratory to examine the molecular factors and mechanisms of tumor budding.

## Abbreviations

CK: cytokeratin
CT: computed tomography
DFS: disease-free survival
EAC: external auditory canal
EMT: epithelial-to-mesenchymal transition
Ln5-γ2: laminin 5-γ2
MCF: middle cranial fossa
MRI: magnetic resonance imaging
SCC: squamous cell carcinoma
